# Impact of a COPD Discharge Care Bundle on Readmissions following Admission with Acute Exacerbation: Interrupted Time Series Analysis

**DOI:** 10.1371/journal.pone.0116187

**Published:** 2015-02-13

**Authors:** Anthony A. Laverty, Sarah L. Elkin, Hilary C. Watt, Christopher Millett, Louise J. Restrick, Sian Williams, Derek Bell, Nicholas S. Hopkinson

**Affiliations:** 1 Department of Primary Care and Public Health, Imperial College London, London, England; 2 Imperial College London NHS Trust, London, England; 3 London Respiratory Team NHS London, London, England; 4 NIHR CLAHRC for Northwest London, London, England; 5 NIHR Respiratory Biomedical Research Unit at Royal Brompton and Harefield NHS Foundation Trust and Imperial College, London, England; University of Athens Medical School, GREECE

## Abstract

**Objectives:**

We evaluated the impact of a COPD discharge care bundle on readmission rates following hospitalisation with an acute exacerbation.

**Design:**

Interrupted time series analysis, comparing readmission rates for COPD exacerbations at nine trusts that introduced the bundle, to two comparison groups; (1) other NHS trusts in London and (2) all other NHS trusts in England. Care bundles were implemented at different times for different NHS trusts, ranging from October 2009 to April 2011.

**Setting:**

Nine NHS acute trusts in the London, England.

**Participants:**

Patients aged 45 years and older admitted to an NHS acute hospital in England for acute exacerbation of COPD. Data come from Hospital Episode Statistics, April 2002 to March 2012.

**Main Outcome Measures:**

Annual trend readmission rates (and in total bed days) within 7, 28 and 90 days, before and after implementation.

**Results:**

In hospitals introducing the bundle readmission rates were rising before implementation and falling afterwards (e.g. readmissions within 28 days +2.13% per annum (pa) pre and -5.32% pa post (p for difference in trends = 0.012)). Following implementation, readmission rates within 7 and 28 day were falling faster than among other trusts in London, although this was not statistically significant (e.g. readmissions within 28 days -4.6% pa vs. -3.2% pa, p = 0.44). Comparisons with a national control group were similar.

**Conclusions:**

The COPD discharge care bundle appeared to be associated with a reduction in readmission rate among hospitals using it. The significance of this is unclear because of changes to background trends in London and nationally.

## Introduction

Chronic Obstructive Pulmonary Disease (COPD) is a common condition, estimated to affect 1.4 million people in England alone [1] and is now the 3^rd^ most common cause of death worldwide[2]. The primary cause of COPD is tobacco smoking and 86% of deaths from COPD are attributable to smoking [3] Acute exacerbations of COPD (AECOPD) are the second most common cause of emergency hospital admission in the UK [3] and a frequent cause of admission with breathlessness. Outcomes are poor with around one third of patients readmitted to hospital within 90 days and an overall 90 day mortality rate of 13.9% [4,5].

There is also considerable variability in outcomes, with 90- day readmission rates ranging between 16% and 48% in the latest European Respiratory Society audit, and ten-fold variation in 90 day[6]. Care bundles have been proposed as an effective approach to improve the quality of patient care. Care bundles are made up of a short series of evidence-based interventions that should be delivered for all patients with a condition, irrespective of ward, or specialty, delivering care. Care bundles have been used in a variety of conditions with effectiveness demonstrated in a range of settings[7–9].

In London, which has a population of 8 million (comparable in size to a number of countries) and a 2008 smoking prevalence of 19%, £100m was spent on COPD care, with more than 90,000 beds used for emergency hospital admissions for patients with a primary diagnosis of COPD in 2008–9 [10]. This unmet patient need, significant use of urgent care and unwarranted variation led to the design and initial implementation of a COPD discharge care bundle by the NIHR CLAHRC for Northwest London which has been described previously [8,11].

The care bundle recommendations are that all patients admitted with an AECOPD receive the following interventions from staff who have the appropriate competencies, most often delivered by one or more clinicians who are members of a respiratory team; (1) Evidence-based smoking cessation interventions (support and pharmacotherapy) and offer of referral to smoking cessation service if a current smoker; (2) Assessment for, explanation of, and referral to, a pulmonary rehabilitation programme; (3) Appropriate education, written personalised information including information about patient support groups (British Lung Foundation (BLF) Breathe Easy Groups), self-management plans (for example, BLF self-management booklet), and, if appropriate, rescue packs for future exacerbations and an oxygen alert card or equivalent; (4) Assessment of patient understanding and use of medications with focus on enabling effective inhaler technique; (5) Leave hospital with booked review once discharged from hospital. Care bundles aim both to improve quality of care, and to standardise delivery, so that all of the included elements are delivered routinely.

The Northwest London COPD Discharge Bundle was endorsed for use across London by the London Respiratory Team, on behalf of the NHS in London, in 2010 and a Commissioning for Quality and Innovation (CQIN) payment framework to support implementation of the COPD discharge bundle by acute trusts was made available for use by commissioners from 2011[10,12]. As a result the bundle was rolled out in a number of acute hospitals in London, incentivised in some by commissioners using the CQIN.

This study aimed to evaluate the impact of implementing the care bundle on readmissions for AECOPD and number of bed days occupied at hospitals using the care bundle. Outcomes for hospitals implementing the care bundle were assessed relative to two comparison groups (1) other NHS trusts in London and (2) all other NHS trusts in England.

## Methods

The care bundle was developed at Chelsea and Westminster NHS Foundation Trust, based on national and international guidelines, and input was sought from the Inner Northwest London Care Community integrated service improvement programme for COPD[8]. After piloting on a respiratory ward at Chelsea and Westminster it was rolled out to three other trusts between April and September 2010. In April 2011, the COPD care bundle was added as a CQUIN for a further five NHS trusts in London (see Table 1).

**Table 1:** Implementation dates of COPD care bundle.

Trust name	Implementation date
Chelsea and Westminster NHS Foundation Trust	October 2009
Imperial College Healthcare NHS Trust	April 2010
West Middlesex University Hospital NHS Trust	May 2010
The North West London Hospitals NHS Trust	September 2010
Epsom and St Helier University Hospitals NHS Trust	April 2011
Kingston Hospital NHS Foundation Trust	April 2011
Mayday Healthcare NHS Trust*	April 2011
St Georges Healthcare NHS Trust	April 2011
The Whittington Hospital NHS Trust**	April 2011

* Subsequently changed to Croydon Health Services NHS Trust

** Merged to become Whittington Health from April 2011

### Data

Data for this study come from Hospital Episode Statistics (HES) which is the national administrative database for hospital activity in England. HES contains information on all admissions to the National Health Service (NHS), and includes clinical information on diagnoses of patients as well as demographic information and the speciality under which they were treated [13]. Data on all COPD admissions to NHS acute trusts utilising the bundle from 1^st^ April 2002 to 31^st^ March 2012 were used. Patients aged 45 years and above were included. All data were analysed at the monthly level, although annualised trends are presented for ease of interpretation. This research was based on the use of anonymous secondary data. The Department of Primary Care & Public Health has approval from the Health and Social Care Information Centre to use this data for health service evaluation.

### Outcome measures

An acute exacerbation of COPD was defined as any patient admitted to hospital as an emergency (*admimeth>21 & <29)* with the ICD-10 codes “J440” or “J441” as their primary reason for admission.

Readmissions among COPD patients were defined as readmission for acute exacerbation of COPD to any of the NHS trusts in the study, within 7, 28 or 90 days of their discharge after an original admission for an acute exacerbation of COPD. The last month of the data series was not used in the analysis of 28 day readmission rates as these could not be calculated, and the last three months were not used for readmission within 90 days. The total number of bed days was calculated by summing the number of nights in hospital for all patients with COPD, whether an original admission or a readmission.

### Analysis

We used an interrupted time series (ITS) approach analysing readmission rates (with Poisson regression using number of admissions as the exposure). The analysis was clustered at the trust level. Time was standardised to time of introduction of the bundle in each trust, to allow for the overall impact of the bundle to be evaluated. In non-bundle trusts, we examined trends on a date when most trusts introduced the bundle, which was October 2009. ITS approaches are considered to be the strongest quasi-experimental approach for evaluating interventions where randomisation isn’t feasible [14] and allow us to estimate separate trends for both intervention and comparison groups both before and after care bundle implementation. Use of ITS allowed examination of differences in COPD readmission trends post intervention, allowing for any trends before its introduction, and including allowing for changes in trends in the control group (i.e. effectively a difference in difference approach). We did not include a term for a step change on introduction of the bundle, as in practice implementation of the bundle was gradual, starting from the date specified. These models are adjusted for the age and sex profile of admitted patients, as well as the month of admission (to take account of seasonal effects) and deprivation level of admitted patients. Deprivation was measured using the Index of Multiple Deprivation (IMD) on the home postcodes of individual patients and divided into thirds based on the national distribution of deprivation ranks. Analysis of bed days used the same model form with linear rather than Poisson regression. All analyses were conducted using Stata version 12.0.

## Results

Analyses of trends in outcomes before and after bundle implementation among trusts which were using the bundle by 2011–12 are shown in Table 2. Trusts using the bundle had a mean of 209.1 7-day COPD readmissions annually (standard deviation (SD) 59.4), and 562.8 (144.9) 28-day readmissions annually. Prior to implementation of the bundle, 7, 28 and 90 day readmissions were rising (e.g. +2.13% for 28-day readmissions) as were number of COPD bed days (+263.7 bed days annually). After implementation of the bundle, all four of these outcomes were declining (e.g. -5.32% pa for 28-day readmissions, p for difference = 0.012).

**Table 2:** Pre/post analysis for bundle trusts.

	7 day readmissions	28 day readmissions	90 day readmissions	Number of bed-days
Mean annual number (SD), 2002–2012	209.1 (59.4)	562.8 (144.9)	1,014 (243.2)	27,769.4 (3,419.4)
Annual trend in COPD admissions pre October 2009^1^	+2.7% (0.019)	+2.1% (0.002)	+1.4% (0.007)	-0.8 (<0.001)
Annual trend in COPD admissions post October 2009^2^	-7.6% (0.028)	-5.3% (0.012)	-1.3% (0.267)	-1.6 (<0.001)

^1^ P-value refers to difference of this trend from zero

^2^ P-values refer to difference between this trend and pre-implementation trend

**Fig 1:**
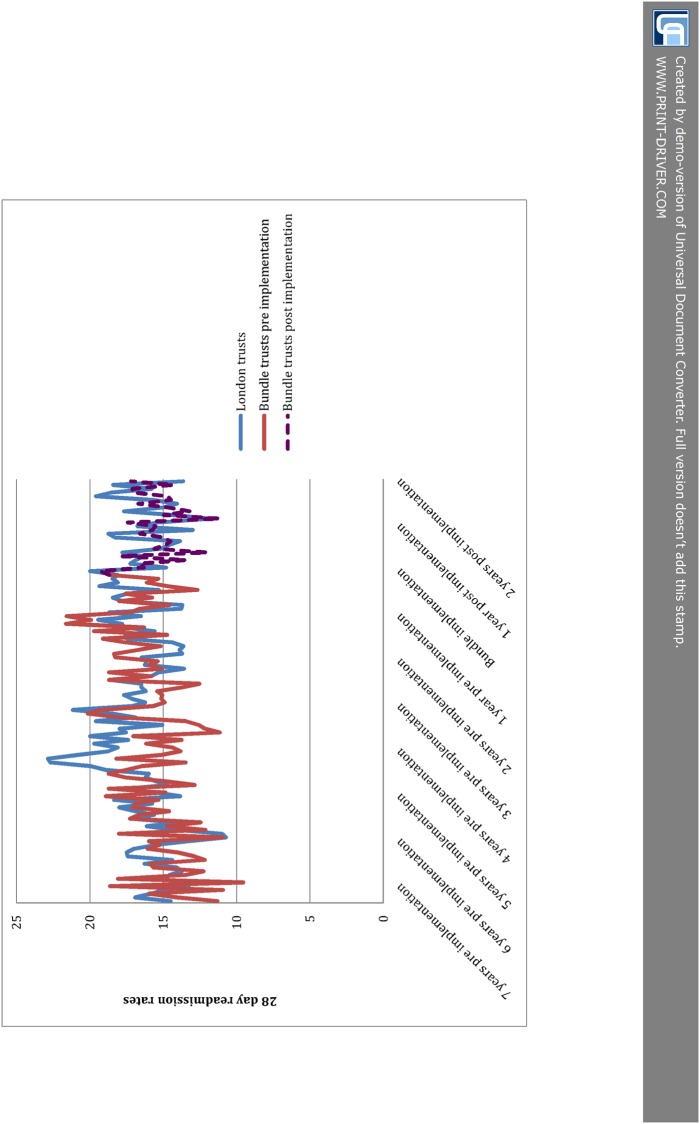
28 day readmissions in bundle trusts and London comparison group.

Results from the ITS analysis comparing bundle trusts to other trusts in London are shown in Table 3. Prior to bundle implementation 7, 28 and 90 day readmissions were rising in both the London comparison group and the bundle trusts. For example, 28-day readmissions were rising at +2.2% per year among bundle trusts compared to +1.3% among other London trusts (p for difference = 0.279). After implementation of the bundle readmissions were coming down among both groups—for 28-day readmissions at-4.6% pa among bundle trusts and-3.2% at London comparison trusts, p for difference (adjusted for baseline) = 0.440. Numbers of bed days were coming down slowly among both groups, before and after implementation of the bundle. Table 3 also shows the effect sizes which would be needed to achieve statistical significance of the care bundle giving the prevailing trends at other London trusts. This shows that given the-3.2% decrease in the rest of London after bundle implementation, the bundle would have had to affect a decrease of 11.7% to be statistically significant at p≤0.05. Fig. 1 gives a graphical representation of the raw data for 28 day readmissions for the bundle trusts as well as the London comparison group. This allows examination of both the variation in the outcome over time and also the lack of clarity over whether the trend for the bundle trusts is different to that for the comparison group.

**Table 3:** Bundle trusts vs. other London trusts for COPD admissions.

	7 day readmissions	28 day readmissions	90 day readmissions	Number of bed-days
Mean annual number for London COPD admissions, 2002–2012	345.3 (49.0)	1,008.4 (141.8)	1,841.6 (207.8)	46,404 (8,130.7)
Mean annual number for bundle COPD admissions, 2002–2012	209.1 (59.4)	562.8 (144.9)	1,014 (243.2)	27,769.4 (3,419.4)
Annual trend in London readmissions pre-implementation ^1^	+1.8% (0.042)	+1.3% (0.008)	+0.7% (0.053)	-1.3 (<0.001)
Annual trend in bundle readmissions pre-implementation ^2^	+3.0% (0.394)	+2.2% (0.279)	+1.4% (0.261)	-2.1 (<0.001)
Annual trend in London readmissions post-implementation ^2^	-4.0% (0.100)	-3.2% (0.035)	-1.9% (0.135)	-2.8 (0.038)
Annual trend in bundle readmissions post-implementation ^3^	-7.3% (0.355)	-4.6% (0.440)	-0.8% (0.884)	-3.6 (0.718)
**Effect size would need for p≤0.05**	**-15.5%**	**-11.7%**	**-10.5%**	**-8.6**

^1^ P-value refers to difference of this trend from zero

^2^ P-values refer to difference between these trends and the trend in London comparison trusts

^3^ P-value refers to difference between this trend and trend in London comparison trusts, adjusted for baseline trends

**Fig 2:**
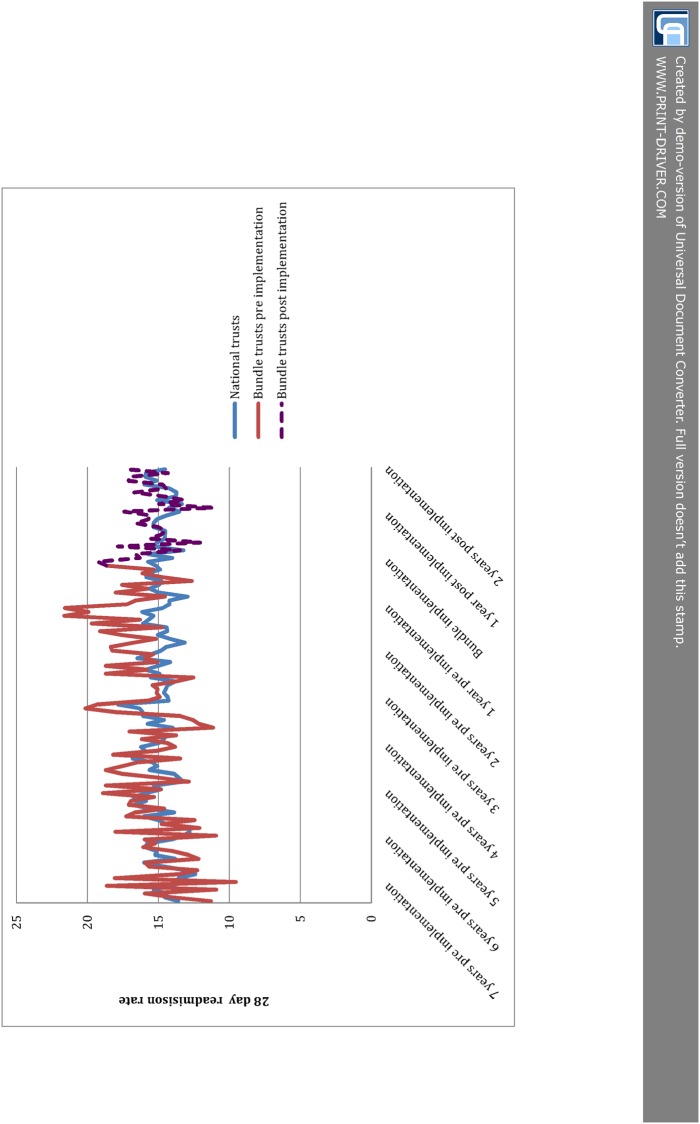
28 day readmissions in bundle trusts and national comparison group.

Results from the ITS analysis comparing bundle trusts to other NHS acute trusts nationally are shown in Table 4. Prior to bundle implementation 7, 28 and 90 day readmissions were rising in both the London comparison group and the bundle trusts, and these rises were faster among bundle trusts than the national comparison group. For example, 28-day readmissions were rising at +2.5% per year among bundle trusts compared to +0.7% among other London trusts (p for difference = 0.012). After implementation of the bundle 7-day readmissions were coming down among both groups at-2.5% pa for the national group and-7.4% pa for the bundle group, p for difference = 0.213. 28 and 90-day readmissions continued to rise among the national comparison group after implementation of the bundle, while these fell for trusts using the bundle. For example, 28-day readmissions +0.41% pa among the national comparison group vs. -4.05% for the bundle group, p for difference = 0.087. After implementation of the bundle, bed days in the bundle group were coming down more quickly than the national comparison group (-1.2 vs. -2.0, p for difference = 0.045). As with the comparison to London, the effect sizes required for statistical significance are large—e.g. 28-day readmission rates would have to have been falling by 4.5%pa to identify an effect against the background change. Fig. 2 gives a graphical representation of the raw data for 28 day readmissions for the bundle trusts as well as the national comparison group.

**Table 4:** Bundle trusts vs. other trusts nationally for COPD admissions.

	7 day readmissions	28 day readmissions	90 day readmissions	Number of bed-days
Mean annual number for national trusts COPD admissions, 2002–2012	3,947.7 (470.1)	11,398.3 (1,189.8)	20,614.9 (2,543.5)	563,526.3 (61,460.4)
Mean annual number for bundle COPD admissions, 2002–2012	209.1 (59.4)	562.8 (144.9)	1,014 (243.2)	27,769.4 (3,419.4)
Annual trend in national readmissions pre-implementation ^1^	+1.4% (<0.001)	+0.7% (<0.001)	+0.3% (0.011)	-1.0 (0.001)
Annual trend in bundle readmissions pre-implementation ^2^	+3.2% (0.126)	+2.5% (0.012)	+1.6% (0.013)	-0.8 (0.029)
Annual trend in national readmissions post-implementation ^2^	-2.5% (<0.001)	+0.4% (0.001)	+1.0% (0.246)	-1.2 (0.001)
Annual trend in bundle readmissions post-implementation ^3^	-7.4% (0.213)	-4.1% (0.087)	-0.9% (0.397)	-2.0 (0.045)
**Effect size would need for p≤0.05**	**-11.7%**	**-4.5%**	**-1.8%**	**N/A**

^1^ P-value refers to difference of this trend from zero

^2^ P-values refer to difference between these trends and the trend in national comparison trusts

^3^ P-value refers to difference between this trend and trend in national comparison trusts, adjusted for baseline trends

S1 to S3 Tables include the results from analyses using a wider definition of COPD of ICD-10 codes J40–44.

## Discussion

### Main findings

The main finding of this study is that the implementation of the COPD discharge care bundle during hospital admission was associated with a change from an upwards trend in readmission rates for patients with COPD to a downwards one. This change however, was not found to be statistically significantly different to either a London or a national comparison group. Due to the small number of NHS trusts using the care bundle and the short time since implementation, large effects on readmission rates would have been needed in order to more confidently assert a positive impact on readmission rates. It is also possible that other work to improve the quality of care in COPD, including systematic approaches to the delivery of the five interventions in the COPD bundle, either individually or as a bundle, in other trusts across London and nationally over this time masked the impact of introducing the bundle [15]. Unfortunately information on these interventions at other trusts was not available for this analysis. The COPD discharge care bundle is recognised by clinicians and commissioners [10,16] as a means to systematise the implementation of high value [17], evidence-based items of care for patients admitted to hospital with COPD which may have a positive impact on readmissions, although further research is still required.

### Significance of the findings

As far as we are aware, this is the first evaluation of the impact of implementation of a COPD discharge care bundle across a number of hospitals in England. The present study develops from our previous work showing a trend towards a decline in hospital COPD readmission rates after the bundle had been implemented on a single respiratory ward [8]. The bundle has been in place for a relatively short period of time so these results may change with more data points. In 5 trusts, implementing the care bundle as part of a CQIN, only 12 months data were available and longer term evaluation would be useful. The advantage of using a CQIN is that it is an enabler for care bundle interventions to be delivered for every patient admitted with AECOPD. This is important in order to reduce unwarranted variation as only 53% of patients admitted with COPD were under the care of a respiratory team at discharge in the last national COPD audit[18]. The CQIN targets set for these hospitals were based on percentage use for all patients admitted for more than 48 hours, whether on a respiratory ward, an acute admissions ward or another medical ward (increasing from 70% to 95% according to local negotiations).

The care bundle approach is helpful in focusing attention on the highest value interventions in COPD care, in particular support and medication to stop smoking and pulmonary rehabilitation, and to highlight deficiencies in the provision of care [17]. Despite the evidence for quit smoking interventions as a very high value treatment in COPD [11], the evidence that for every 1% increase in smoking prevalence in the COPD population there is a 1% increase in admission rates for COPD [19], smoking prevalence in patients admitted to hospital with COPD in England in 2008 was 33%[18]. In the UK, many areas still have limited and only some or no provision of pulmonary rehabilitation for patients with COPD[20,21], including some areas in London. This is despite a Grade A evidence base for pulmonary rehabilitation for people who are breathless due to COPD and clinical guidelines that reflect this [22–24] including meta-analysis suggesting that post exacerbation pulmonary rehabilitation has an estimated number needed to treat of four to prevent one readmission [25]. The institution of a care bundle brings these unmet population needs into focus and may help to guide commissioning. The British Thoracic Society are currently trialling a version of the care bundle, including additional elements when patients are admitted to hospital, such as ensuring a correct diagnosis of COPD [11,26]. Results from this evaluation are likely to impact on use of care bundles. Research on implementation of the bundle has concluded that successful implementation relied on an awareness of the likely challenges of implementation, which include a lack of staff engagement, issues of clinical coding and the added workload of the bundle for frontline staff [11].

A recent audit of admissions with AECOPD found there to be wide variability in adherence to quality standards across Europe[6], and these results suggest that using a care bundle may be an effective method of standardising and improving care. Hospital admission is only one of a number of opportunities and locations to deliver high value COPD care and a population based approach, including locally enhanced services for COPD, has also been shown to have a significant impact on patient care [27,28] particularly when part of a shared approach across the whole pathway[29].

### Methodological issues

This study has the weaknesses inherent in using routine data for evaluation. Due to the short period of time since bundle implementation and the small number of trusts using the bundle, estimation of post-intervention trends was subject to random measurement error. This means that very large effects would have to be associated with the bundle in order to be found to be statistically significant with this data. Other weaknesses also include concerns over the accuracy of clinical coding (upon which case ascertainment was based), although evaluation of coding by the Audit Commission have found it to be valid [30]. Sensitivity analyses using a wider definition of diagnostic codes for COPD gave similar results. We limited inclusion to patients with ICD-10 codes for an acute exacerbation of COPD, and it is likely that some patients admitted with COPD were not picked up using these specific codes.

Our analysis only included patients with a primary diagnosis of COPD (considered as the reason for admission) in the COPD group. This was because patients with secondary diagnoses of COPD are likely to have been admitted to hospital for reasons other than COPD, and outside the scope of the care bundle. It is difficult to ascertain whether COPD may have contributed to their hospital admission, or whether it was coded as a pre-existing condition only. However, this does not take account of the group of patients admitted with pneumonia who have COPD where their underlying COPD is directly contributory.

We did not include a measure of the number or extent of comorbid conditions which patients may have had, and have only controlled for their age, sex and deprivation level. There are two reasons for this. Firstly, the population develops increasing comorbidities over time, so adjusting for this would be likely to make the effect of the bundle more positive. Secondly, many of the comorbidities at a patient level should already be picked up by controlling for age, sex and deprivation. The approach we have taken is therefore a more conservative estimation of the effect of the care bundle. However, it is well recognised that for patients with COPD only a proportion of readmissions are with an AECOPD and evaluating the impact of interventions in one disease domain may be less useful in the longer term than evaluating impact on any readmissions and total bed-days over a year.

We compared COPD patient readmission rates among trusts using the care bundle to two comparison groups which were not to our knowledge using care bundles systematically for COPD. During the time course of the study other changes may have influenced readmission rates including changes in community care or quality of primary care. Additionally, data was not available for this study on whether some patients were offered only part of the package of measures in the bundle, or what take-up of interventions such as pulmonary rehabilitation was. This study focused on readmissions to hospital, rather than other measures, such as mortality, which have been criticised as being too variable to be a useful metric of quality of care [4]. As a strategy designed to systematise the delivery of evidence based interventions other measures of patient experience may be more sensitive and could be considered for future study. Further work could also examine the cost-to-benefits of using such care bundles.

### Conclusion

The COPD discharge care bundle appeared to be associated with a reduction in readmission rate among hospitals using it. Caution is needed in evaluating this however given a background downward trend in readmission rates in London and nationally which make determining the effect of the care bundle intervention more difficult.

## Supporting Information

S1 Table Pre/post analysis for bundle trusts, using ICD-10 codes J40–44. (DOCX)

S2 Table Bundle trusts vs. other London trusts for COPD admissions, using ICD-10 codes J40–44. (DOCX)

S3 Table Bundle trusts vs. other trusts nationally for COPD admissions, using ICD-10 codes J40–44. (DOCX)
