# Supplementary material for: Impact of a COPD Discharge Care Bundle on Readmissions following Admission with Acute Exacerbation: Interrupted Time Series Analysis
Source: PLoS One. 2015 Feb 13;10(2):e0116187. doi: 10.1371/journal.pone.0116187 (PMC4332682; doi:10.1371/journal.pone.0116187)
Supplement: S1 Table — (DOCX) [file pone.0116187.s001.docx]

**Table S1: Pre/post analysis for bundle trusts, using ICD-10 codes J40-44**

|  | 7 day readmissions | 28 day readmissions | 90 day readmissions | Number of bed-days |
| --- | --- | --- | --- | --- |
| Mean annual number (SD), 2002 - 2012 | 272.2 (69.7) | 727.3 (163.9) | 1,335.5 (284.4) | 38,021.2 (4,528.9) |
| Annual trend in COPD admissions pre October 2009^1^ | +1.6% (0.097) | +1.4% (0.020) | +0.9% (0.034) | -0.9 (0.001) |
| Annual trend in COPD admissions post October 2009^2^ | -6.6% (0.047) | -4.3% (0.031) | -1.0% (0.376) | -1.8 (0.010) |

^1^ P-value refers to difference of this trend from zero

^2^ P-values refer to difference between this trend and pre-implementation trend
