# Supplementary material for: Impact of a COPD Discharge Care Bundle on Readmissions following Admission with Acute Exacerbation: Interrupted Time Series Analysis
Source: PLoS One. 2015 Feb 13;10(2):e0116187. doi: 10.1371/journal.pone.0116187 (PMC4332682; doi:10.1371/journal.pone.0116187)
Supplement: S2 Table — (DOCX) [file pone.0116187.s002.docx]

**Table S2: Bundle trusts vs. other London trusts for COPD admissions, using ICD-10 codes J40-44**

|  | 7 day readmissions | 28 day readmissions | 90 day readmissions | Number of bed-days |
| --- | --- | --- | --- | --- |
| Mean annual number for London COPD admissions, 2002 - 2012 | 454.1 (56.3) | 1,353.1 (175.1) | 2,472.2 (248.4) | 63,277.9 (12,281.9) |
| Mean annual number for bundle COPD admissions, 2002 - 2012 | 272.2 (69.7) | 727.3 (163.9) | 1,335.5 (284.4) | 38,021.2 (4,528.9) |
| Annual trend in London readmissions pre-implementation ^1^ | +1.4% (0.063) | +0.6% (0.173) | +0.2% (0.522) | -1.7 (<0.001) |
| Annual trend in bundle readmissions pre-implementation ^2^ | +1.9% (0.700) | +1.5% (0.238) | +1.0% (0.173) | -1.0 (<0.001) |
| Annual trend in London readmissions post-implementation ^2^ | -4.4% (0.058) | +4.8% (0.025) | +2.7% (0.105) | -1.2 (0.666) |
| Annual trend in bundle readmissions post-implementation ^3^ | -6.9% (0.487) | -3.8% (0.001) | -0.5% (0.093) | -1.4 (0.671) |
| **Effect size would need for p≤0.05** | **-19.3%** | **-2.2%** | **-0.6%** | **-6.5** |

^1^ P-value refers to difference of this trend from zero

^2^ P-values refer to difference between these trends and the trend in London comparison trusts

^3^ P-value refers to difference between this trend and trend in London comparison trusts, adjusted for baseline trends
