# Supplementary material for: Impact of a COPD Discharge Care Bundle on Readmissions following Admission with Acute Exacerbation: Interrupted Time Series Analysis
Source: PLoS One. 2015 Feb 13;10(2):e0116187. doi: 10.1371/journal.pone.0116187 (PMC4332682; doi:10.1371/journal.pone.0116187)
Supplement: S3 Table — (DOCX) [file pone.0116187.s003.docx]

**Table S3: Bundle trusts vs. other trusts nationally for COPD admissions, using ICD-10 codes J40-44**

|  | 7 day readmissions | 28 day readmissions | 90 day readmissions | Number of bed-days |
| --- | --- | --- | --- | --- |
| Mean annual number for national trusts COPD admissions, 2002 - 2012 | 5,181.8 (529.5) | 15,092.9 (1,307.8) | 27,788.9 (3,146.6) | 745,409.3 (88,535.4) |
| Mean annual number for bundle COPD admissions, 2002 - 2012 | 272.2 (69.7) | 727.3 (163.9) | 1,335.5 (284.4) | 38,021.2 (4,528.9) |
| Annual trend in national readmissions pre-implementation ^1^ | +1.4% (<0.001) | +0.6% (<0.001) | 0.3% (0.006) | -1.1 (<0.001) |
| Annual trend in bundle readmissions pre-implementation ^2^ | +2.1% (0.507) | +1.6% (0.089) | +1.1% (0.076) | -1.0 (0.175) |
| Annual trend in national readmissions post-implementation ^2^ | -3.9% (<0.001) | +1.5% (0.085) | +1.8% (0.001) | -1.2 (0.049) |
| Annual trend in bundle readmissions post-implementation ^3^ | -7.2% (0.267) | -3.8% (0.007) | -1.2% (0.052) | -1.9 (0.103) |
| **Effect size would need for p≤0.05** | **-12.7%** | **-2.8%** | **-1.21%** | **-2.3** |

^1^ P-value refers to difference of this trend from zero

^2^ P-values refer to difference between these trends and the trend in national comparison trusts

^3^ P-value refers to difference between this trend and trend in national comparison trusts, adjusted for baseline trends
